# Chemical composition and antimicrobial activity of the essential oils in different populations of *Coriandrum sativum* L. (coriander) from Iran and Iraq

**DOI:** 10.1002/fsn3.4047

**Published:** 2024-02-20

**Authors:** Seyed Mehdi Talebi, Abbas Naser, Mansour Ghorbanpour

**Affiliations:** ^1^ Department of Biology, Faculty of Science Arak University Arak Iran; ^2^ Department of Medicinal Plants, Faculty of Agriculture and Natural Resources Arak University Arak Iran

**Keywords:** chemotype, infraspecific diversity, photogenes, phytochemistry, volatile fraction

## Abstract

Coriander (*Coriandrum sativum* L.) is an annual herb belonging to the Apiaceae family that is grown worldwide. This aromatic herb has been used for its nutritional value and biological properties. In this study, we compared the essential oil composition and antibacterial activity of coriander seeds from nine Iranian and Iraqi populations for the first time. The seed oils were extracted using a Clevenger‐type apparatus, and their chemical composition was determined using GC and GC/MS Agilent apparatuses. The antimicrobial activity of the oils was tested against three infectious bacteria (*Staphylococcus aureus*, *Escherichia coli*, and *Pseudomonas aeruginosa*) using the agar well diffusion method. The experiments were repeated three times, and the results were analyzed using PAST, SAS, and SPSS software. The results showed that oxygenated monoterpenes, especially linalool, were the major compounds in the oils, followed by α‐pinene, γ‐terpinene, and geranyl acetate. The proportions of these compounds varied among the populations. Trace amounts of other compounds were also detected, some of which were only found in certain populations. The populations were detected as linalool chemotype, and classified into four groups based on their chemical constituents in the UPGMA tree. The PCA‐Biplot showed that these groups were characterized by the presence and percentage of specific compounds. The essential oils showed bacterial growth inhibitory properties only at 100% concentration. *S. aureus* was the most sensitive bacterium to the coriander essential oil, while the essential oils of all populations inhibited the growth of this bacterium. Additionally, the essential oils were more effective than antibiotics against *E. coli*. These findings contribute to our understanding of coriander seed essential oil by providing data on antibacterial activity and chemical characteristics. Furthermore, the study highlights the importance of selecting populations based on their specific essential oil profiles for antibacterial applications.

## Graphical abstract

In this study, the seed essential oil composition and its antibacterial activities were evaluated in nine Iranian and Iraqi coriander populations. Results indicated that the oxygenated monoterpenes constituted a great proportion of the oils, with linalool as the major compound, followed by α‐pinene, γ‐terpinene, and geranyl acetate as other main compounds.

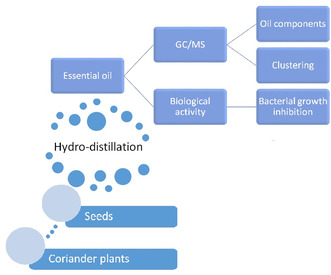

## 1 INTRODUCTION

*Coriandrum sativum* L., which is known as coriander, is an aromatic glabrous annual herb of the Apiaceae/Umbelliferae family and is widely utilized for its nutritional and medicinal properties. This plant originated in the European‐Mediterranean phytogeographic region and was then distributed all over the world (Afshari et al., 2023; Mahleyuddin et al., 2022). The name of this species is derived from the Greek word Korion, which means bug (Shoaib et al., 2023). Two types of coriander herbal materials are used: fresh leaves and mature seeds. The coriander leaves are utilized as a flavoring agent and adjuvant in the preparation of diverse foods, preservation, and prevention of foodborne diseases and food spoilage. Coriander seeds are added to foods and salads as an aromatic spice; moreover, they act as digestive agents that accelerate food digestion (Mandal & Mandal, 2015). Coriander is an aromatic herb that contains essential oils, flavonoids, phenolics, and alkaloid compounds. The phenolic and flavonoid compounds in coriander have antioxidant and anti‐inflammatory properties and are also known for their anticancer and neuroprotective effects. On the other hand, the alkaloid and terpene compounds present in coriander are helpful in managing diabetes and hypertension, making it a popular choice in both traditional and modern medicine. Coriander is known to effectively treat a variety of conditions in the urinary, skin, cardiovascular, digestive, and respiratory systems. It is also used to cure diabetes, dyspepsia, flatulence, and diarrhea diseases (Pandey et al., 2023; Scandar et al., 2023).

The study of the coriander essential oil composition and its biological activities against pathogens is the subject of several investigations around the world (Asgarpanah & Kazemivash, 2012; Ashraf et al., 2019; Kubo et al., 2004; Saeed & Tariq, 2007; Sambasivaraju & Za, 2016; Silva et al., 2011; Zardini et al., 2012). These studies indicated that linalool, an oxygenated monoterpene compound, is the major part of the essential oil of seeds, while others such as pinene, terpinene, nerol, and geranyl acetate have a notable percentage. In addition, its essential oil shows high antibacterial activity against some important bacteria, including: *Staphylococcus aureus*, *Escherichia coli*, *Klebsiella pneumoniae*, *Salmonella typhimurium*, *Salmonella choleraesuis*, *Bacillus subtilis*, *Pseudomona*s *aeruginosa*, and *Acinetobacter baumannii*.

It has been suggested that the essential oil compounds found in coriander are responsible for its biological activities, such as antioxidant, anti‐inflammatory, and anticancer properties (Pandey et al., 2023; Shoaib et al., 2023). In this study, we examined the composition of essential oils and their antibacterial activity against *P. aeruginosa*, *E. coli*, and *S. aureus* in nine populations of *C. sativum* from Iran and Iraq. Our objectives were to: (1) compare the essential oil constituents between Iranian and Iraqi populations, (2) identify possible chemotypes for future breeding programs, and (3) evaluate the antibacterial activity of the oils against the aforementioned bacteria. To our knowledge, this is the first study to compare the essential oil of coriander seeds in this manner.

## 2 MATERIALS AND METHODS

### 2.1 Plant samples and essential oils extraction

**FIGURE 1:**
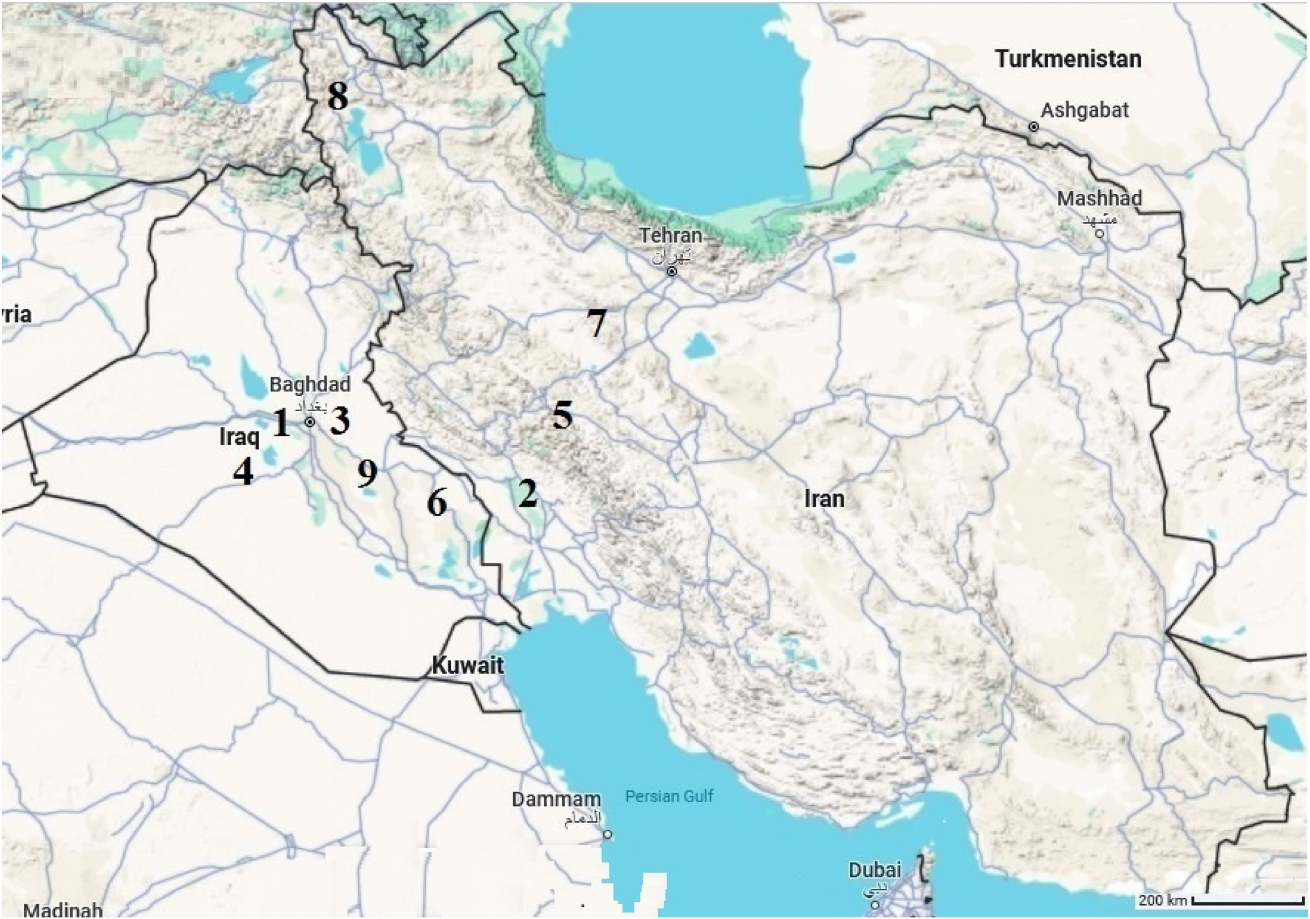
Distribution map of the investigated Iranian and Iraqi coriander populations (numbers indicate the population code based on Table 1).

For this study, seeds of nine coriander populations were collected from the cultivated fields in different regions of Iran (four populations) and Iraq (five populations) during autumn 2022 (Figure 1). The population name and address are presented in Table 1. Per population, 1000 g of healthy, intact, and mature seeds were cleaned and grounded. Then, their essential oils were extracted by the hydro‐distillation method using a Cleavinger‐type apparatus for 3 h. We repeated the oil extraction three times.

**TABLE 1:** The name and location of the evaluated populations collected from the cultivated fields in Iran and Iraq.

No.	Localities	Altitude	Longitude	Elevation
1	Iraq, Baghdad governorate, Baghdad	33°31′	44°36′	34 m
2	Iran, Khuzestan province, Dezful	32°38′	48°39′	150 m
3	Iraq, Diyala governorate, Diyala	33°73′	45°19′	100 m
4	Iraq, Karbala governorate, Karbala	32°60′	44°01′	28 m
5	Iran, Lorestan Province, Khoram Abad	33°46′	48°33′	1200 m
6	Iraq, Maysan governorate, Amareh	31°83′	47°14′	10 m
7	Iran, Markazi province, Saveh	35°02′	50°35′	1000 m
8	Iran, West Azerbaijan, Urmia	37°55′	45°07′	1400 m
9	Iraq, Wasit governorate, Kut	32°40′	45°45′	40 m

### 2.2 Antimicrobial activity with well diffusion method

The biological activities of the essential oils were examined using the agar well diffusion method. The bacterial strains of *E. coli* IBRC‐M 11018 (ATCC 25922), *P*. aeruginosa IBRC‐M10828 (ATCC 10145) as Gram‐negative bacteria, and *S. aureus* IBRC‐M 10690 (ATCC 33591) as Gram‐positive bacteria were incubated in Tryptone Soya Agar (TSA, Sigma‐Aldrich) at 37°C for 24 h. We prepared microbial suspensions of these bacteria in saline water (0.9%) with 0.5 McFarland turbidity standards, then they were streaked on Mueller Hinton Agar (MHA) using the sterile swabs. After solidification of the media, we cut five wells, each 4 mm in diameter, in agar. Based on the National Committee for Clinical Laboratory Standards (NCCLS, 2003) advice, we used a broth microdilution approach to determine the minimum inhibitory concentration (MIC). It is the minimum concentration of an antibacterial agent, in mg/L or μg/mL, that, under strictly controlled in vitro conditions, completely inhibits the visible growth of the examined strain of an organism (Kowalska‐Krochmal & Dudek‐Wicher, 2021). Then, according to previous investigations (Kozłowska et al., 2018; Rattanachaikunsopon & Phumkhachorn, 2010), 100 μL of essential oils in concentrations of 100%, 10%, and 1% (v/v) and antibiotic discs were placed in wells to examine their inhibitory effects against the growth of the tested bacteria. We used vancomycin 30 μg (for *S. aureus*) and ciprofloxacin 5 μg (for *E. coli* and *P. aeruginosa*) antibiotics as the positive controls, and water for the negative control. We placed the inoculated Mueller‐Hinton agar media at 4°C for 2 h and then incubated at 37°C for 24 h. Refrigeration of the inoculated plates slows down the bacteria growth and allows the essential oil solutions to diffuse into the agar media. The experiment was carried out three times, and the antibacterial activity was examined by measuring the growth inhibition zone for each well, and the average values were taken.

### 2.3 GC and GC–MS analyses

We performed the GC‐FID analysis of the oils on a ThermoQuest‐Finnigan apparatus equipped with a DB‐5 fused silica column in the size of 60 m × 0.25 mm i.d. and a film thickness of 0.25 μm. We utilized nitrogen as the carrier gas at a constant flow rate of 1.1 mL min^−1^. The split ratio was 1:50. The initial temperature of the oven was 60°C, then increased to 250°C at a rate of 5°C min^−1^. The injector temperature was kept at 250°C, followed by the detector temperature at 280°C. We performed the GC–MS analysis on a ThermoQuest‐Finnigan Trace GC–MS apparatus that was equipped with a DB‐5 fused silica column (60 m × 0.25 mm i.d. and a film thickness of 0.25 μm). The oven temperature was 60°C and raised to 250°C at a rate of 5°C min^−1^. The apparatus used helium as the carrier gas at a flow rate of 1.1 mL min^−1^ with a split ratio of 1:50. We determined the composition of essential oils by detecting compound retention indices under temperature‐programmed conditions for *n*‐alkanes and the essential oils on a DB‐5 column. For the detection of essential oil constituents, we compared the mass spectra of constituents with those that existed in the internal reference mass spectra library or with authentic constituents, and confirmed this by comparing the retention indices of compounds with those of authentic constituents or those that are available in valuable references (e.g., Adams, 2007).

### 2.4 Statistical data analysis

Data were subjected to one‐way ANOVA test using the SAS‐Statistical Analysis System‐release 9.4M6, SAS/STAT 15.1 (2018) (SAS Institute, Cary, NC, USA). The normality of all data was preliminary checked using the Shapiro–Wilk normality test. Relationships among the populations were evaluated by principal component analysis (PCA) using SPSS ver. 26 software (Statistical Package for the Social Sciences, IBM Institute, New York, USA). It is a dimensionality reduction method that is often utilized to decrease the dimensionality of large data sets by transforming a large set of variables into a smaller one that still contains most of the information in the large set. The analysis reveals the eigenvalues and eigenvectors of the variance–covariance matrix or the correlation matrix. The eigenvalues, giving a measure of the variance accounted for by the corresponding eigenvectors (components), are displayed together with the percentages of variance accounted for by each of these components (Jolliffe & Jorge, 2016). Hierarchical cluster analysis (HCA) was employed to place the populations into groups with similar properties (Herdman et al., 2011). For HCA, we performed the unweighted pair group method with arithmetic mean analysis (UPGMA) by Euclidean distance method using PAST ver. 4.15 software (PAleontological Statistics‐University of Oslo, Oslo, Norway). This analysis is arguably the most popular HCA method in use, remains widely cited, and is extremely popular. UPGMA is conceptually easy to understand and fast in practice, an important consideration as big data sets are becoming the norm in many areas (Moulton et al., 2018). UPGMA is a distance method that needs a distance matrix. Therefore, a similarity matrix is calculated. Then, similarity coefficients were transformed into distances. Three replications were done for each antibacterial activity measurement.

## 3 RESULTS

### 3.1 Essential oil composition

The essential oil composition of the examined populations has been summarized in Table 2. The plant samples have been harvested from the cultivated fields, and a high variation was detected in essential oil yield among the populations. A total of more than 98% of the essential oil composition was identified. The predominant fraction of the essential oils consisted of oxygenated monoterpenes, accounting for 70 (Urmia population) to 79% (Baghdad population), while monoterpene hydrocarbons made up 20.05 (Baghdad population) to 29.35% (Urmia population) of the oils in all the tested populations. No sesquiterpene hydrocarbons or oxygenated sesquiterpene compounds were detected. However, a trace amount (0.61%–1.78%) of other compounds (such as aldehydes and hydrocarbons) was detected.

**TABLE 2:** Essential oil composition of the evaluated Iranian and Iraqi coriander populations (RT: Retention Time and KI: Kovats Index).

Components	RT	KI	Iraqi populations					Iranian populations			
			Baghdad	Diyala	Karbala	Maysan	Wasit	Dezful	Lorestan	Saveh	Urmia
α‐Thujene	11.19	930	–	0.1	0.1	0.08	–	0.13	–	–	0.13
α‐Pinene	11.58	939	8.31	11.91	10.45	10.2	13.42	9.42	9.23	9.07	12.7
Camphene	12.46	954	0.61	0.11	0.11	0.23	0.18	0.13	–	–	–
Sabinene	13.64	975	0.29	0.36	0.36	0.31	0.21	0.43	0.28	0.29	0.48
ß‐Pinene	13.91	979	0.58	0.89	0.83	0.81	0.86	0.8	0.76	0.78	1.17
ß‐Myrcene	14.48	991	0.54	0.3	0.28	0.36	0.17	0.29	0.25	0.27	0.31
o‐Cymene	16.47	1026	1.7	1.45	1.52	1.24	1.72	2.2	1.47	1.51	3.58
Limonene	16.62	1029	1.46	0.27	0.25	0.64	2.92	0.21	0.2	0.23	0.44
γ‐Terpinene	18.17	1060	6.27	10	9.8	8.79	4.41	7.09	8.18	8.75	10.54
Octanol	18.98	1068	–	–	–	0.15	–	0.61	1.11	0.54	0.42
α‐Terpinolene	19.55	1089	0.29	–	–	0.1	–	–	–	–	–
Linalool	20.50	1097	74.14	70.09	70.6	71.97	64.44	74.15	74.09	73.22	63.77
Nonanal	20.76	1100	–	–	–	–	–	0.23	0.17	–	0.19
Camphor	23.02	1146	2.19	0.33	0.33	0.74	0.6	0.34	0.29	0.28	–
Borneol	24.23	1169	0.45	–	–	0.15	–	–	–	–	–
Terpinen‐4‐ol	24.57	1177	0.18	0.11	0.11	0.14	–	0.14	–	–	0.14
α‐Terpineol	25.35	1189	0.21	–	–	0.22	–	–	–	–	–
Decanal	25.75	1201	–	0.21	–	–	0.17	–	0.27	0.26	–
Citronellol	26.70	1225	–	–	0.14	–	0.15	0.16	–	–	–
Geraniol	27.83	1253	0.4	0.2	0.25	0.34	–	–	–	–	–
cis‐Dihydrocarvone	25.55	1193	–	–	–	–	0.44	–	–	–	–
trans‐Dihydrocarvone	25.86	1201	–	–	–	–	0.77	–	–	–	–
Carvotanacetone	27.86	1247	–	–	–	–	1.61	–	–	–	0.47
Nerol	27.87	1229	–	–	–	–	–	0.29	0.2	0.21	–
Geranyl acetate	33.50	1381	2.36	2.79	2.79	2.97	4.73	2.99	2.73	–	5.65
Apiole	43.67	1678	–	–	–	–	0.24	–	–	–	–
Nonadecane	53.03	1900	–	–	–	–	0.79	–	–	–	–
Heneicosane	59.37	2100	–	–	–	–	0.57	–	–	–	–
Dodecanal	37.46	1466	–	0.75	1.13	0.42	1.46	0.23	–	0.82	–
Monoterpene hydrocarbons			20.05	25.39	23.7	22.76	23.89	20.07	20.37	20.90	29.35
Oxygenated monoterpenes			79.93	73.52	74.22	76.31	74.44	78.07	77.31	77.35	70.03
Sesquiterpene hydrocarbons			–	–	–	–	–	–	–	–	–
Oxygenated sesquiterpenes			–	–	–	–	–	–	–	–	–
Other				0.96	1.13	0.81	1.53	0.97	1.78	1.62	0.61

Among the major oil components, linalool was the most abundant in the range of 63.77 (Urmia population) to 74.15% (Baghdad and Dezful populations), followed by α‐pinene, whose highest and lowest amounts were detected in Baghdad (8.31%) and Urmia (12.7%) populations, respectively. γ‐Terpinene was the third major component in the evaluated essential oils, and its percentages varied more than twice among the populations. Additionally, geranyl acetate was detected as the fourth main compound in all the populations, except for the Saveh population, which contained o‐cymene as the fourth major compound. Several trace amounts of compounds were detected in the examined essential oils. Among them, a few compounds (such as sabinene, β‐pinene, β‐myrcene, and limonene) were general and observed in all, and some others (e.g., α‐thujene, camphene, and terpinen‐4‐ol) were detected in most populations. Meanwhile, some of the trace amount compounds had a restricted existence and were detected in one or two populations, for instance, heneicosane, apiole, cis‐dihydrocarvone, trans‐dihydrocarvone, carvotanacetone, α‐terpineol, and α‐terpinolene.

### 3.2 Population clustering

**FIGURE 2:**
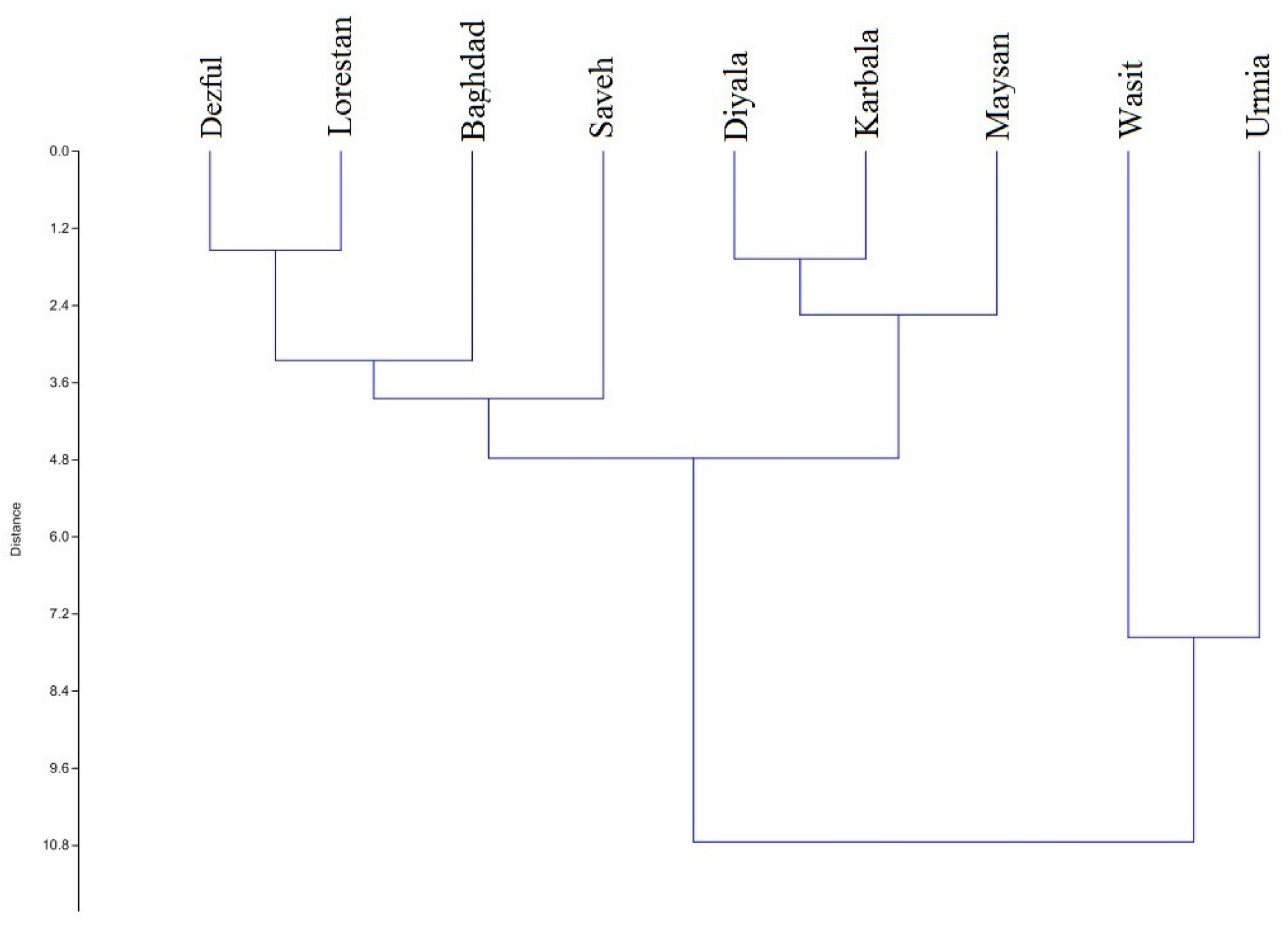
The UPGMA HCA dendrogram of the examined coriander populations based on the essential oil composition.

**FIGURE 3:**
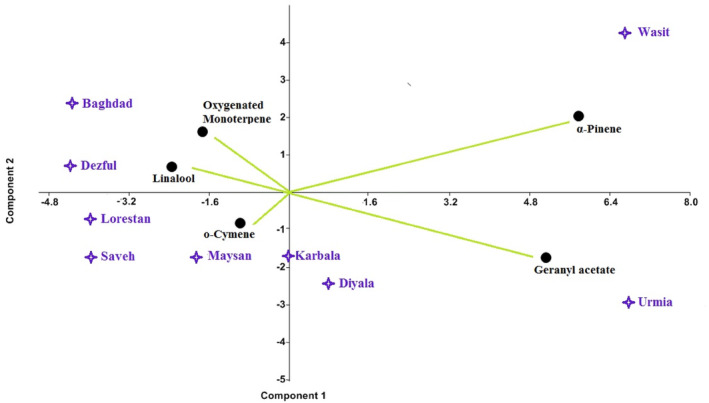
The PCA‐biplot of the essential oil composition in the evaluated coriander populations.

The evaluated populations were linalool chemotype, which clustered into four main groups according to the UPGMA tree constructed on the seed essential oil composition (Figure 2). In this sense, the Wasit and Urmia populations were placed far from the others and characterized as two speared groups. Maysan, Karbala, and Diyala populations were grouped closely as a group, and the rest of the populations (Dezful, Lorestan, Baghdad, and Saveh) stood close together as another group. Similar results were obtained by the PCA‐biplot, where component no. 1 acted as a cut factor and divided the populations into two main clusters, and then component no. 2 divided these clusters into some sub‐groups (Figure 3). The PCA analysis indicated that the first two components contained more than 91% of the total variances (Table 3). According to this plot, some of the identified groups were characterized by special chemical compounds. For example, the amounts of geranyl acetate and α‐pinene were the distinguishing variables for identifying Wasit and Urmia populations. The Maysan population was distinguished by the o‐cymene percentage. Additionally, the Dezful and Baghdad populations had the highest amount of oxygenated monoterpene.

**TABLE 3:** PCA analysis to explore the components and their variance.

Principal components	Eigen value	% variance
1	21.54	74.25
2	4.93	17.00
3	1.38	4.74
4	0.53	1.83
5	0.43	1.48
6	0.11	0.37

### 3.3 Antimicrobial activity of essential oils

**FIGURE 4:**
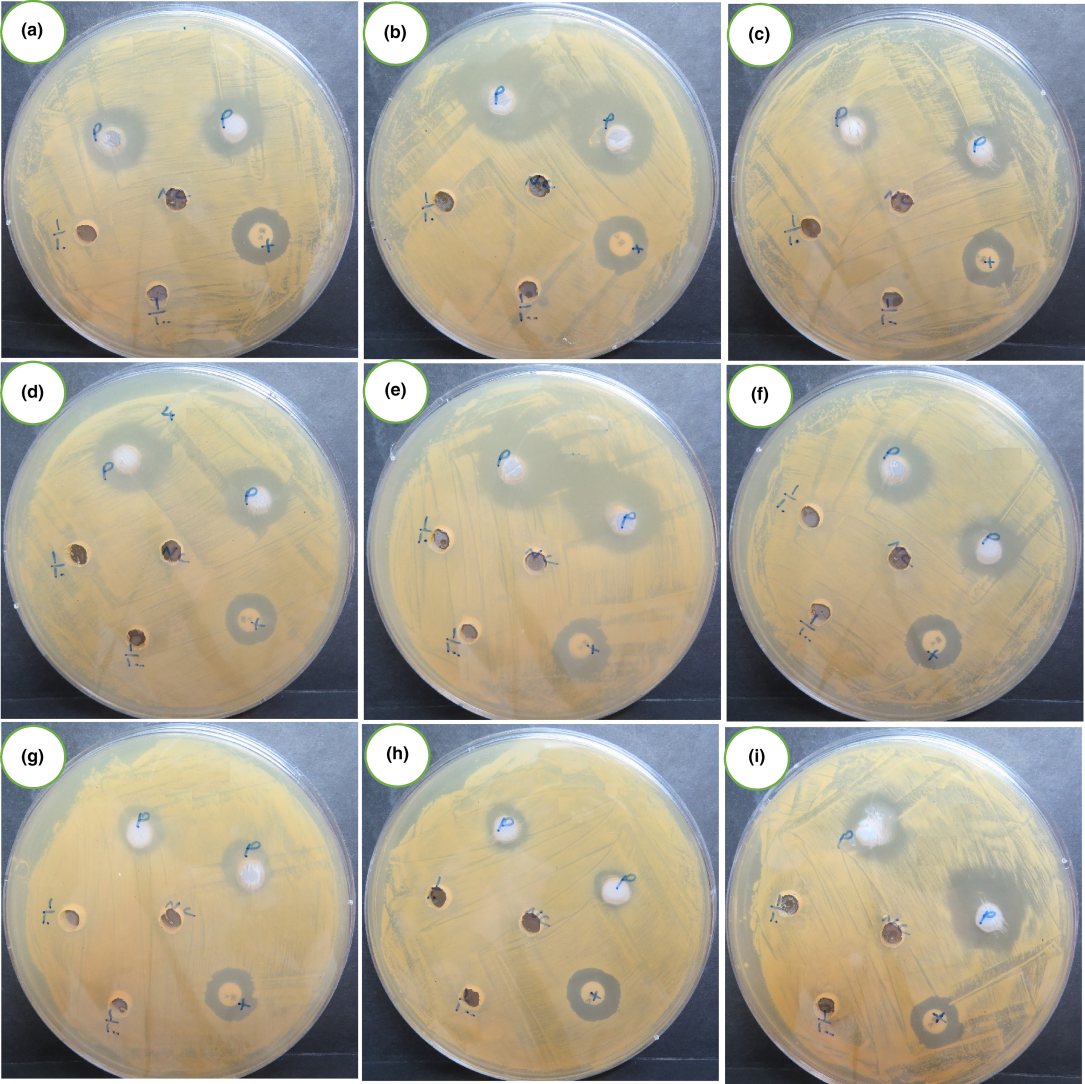
Image of the antibacterial study of the tested populations against *S. aureus*. a–i, population code according to Table 1; the central hole (NC) indicates the negative control; P, shows the essential oil in 100%; two left holes (1/10 and 1/100) reveal the essential oils in 10% and 1%, respectively; and X explores the related antibiotic (ciprofloxacin).

The biological activity of the essential oils extracted from the evaluated populations was tested against three distinct bacterial strains: *P. aeruginosa*, *E. coli*, and *S. aureus* (Figure 4). For all the populations, essential oils in concentrations of 1% and 10% did not exhibit antibacterial activity, and the detected biological activity belonged to the concentration of 100% (Table 4).

**TABLE 4:** Diameter of growth inhibitory zones after 24 h incubation at 37°C.

Populations	*P. aeruginosa*		*E. coli*		*S. aureus*	
	Oil	Ciprofloxacin	Oil	Ciprofloxacin	Oil	Vancomycin
Baghdad	15 ± 1.5	25	9 ± 0.5	8	15 ± 2.0	13
Dezful	11 ± 0.4	24	13 ± 0.6	8	20 ± 0.5	12.5
Diyala	0	25	11 ± 0.5	9	13 ± 2.2	13.5
Karbala	10 ± 2.0	25	13 ± 0.5	8	17 ± 1.0	13
Lorestan	13 ± 1.0	25	14 ± 0.4	9	20 ± 0.4	13.5
Maysan	0	25	17 ± 0.8	9	16 ± 0.1	14.0
Saveh	12 ± 0.6	25	18 ± 2.0	11	13 ± 1.2	14.0
Urmia	0	25	13 ± 0.7	9	12 ± 2.0	14.0
Wasit	11 ± 1.5	25	15 ± 2.00	9	17 ± 1.5	13.0

*Note*: All values are in mm.

The essential oil of the Baghdad population demonstrated a notable inhibitory effect on the growth of all the bacterial strains. Essential oils of the Dezful, Karbala, and Wasit populations inhibited the growth of *P. aeruginosa* and *E. coli* bacteria; however, their highest inhibitory zones were detected for *S. aureus*. In the Diyala and Urmia populations, oils did not affect *P. aeruginosa* growth. Meanwhile, these oils exhibited inhibitory effects against *S. aureus* and *E. coli* growth. The essential oil of the Lorestan population significantly inhibited the growth of *E. coli* and *P. aeruginosa*. Meanwhile, this oil demonstrated a substantial effect on *S. aureus*. Seed essential oil obtained from the Maysan population exhibited no growth inhibitory effects against *P. aeruginosa* growth. However, its highest inhibitory effect was registered against *E. coli*. In the Saveh population, essential oil displayed a moderate inhibitory effect against *S. aureus* and *P. aeruginosa* but substantial inhibitory effects against *E. coli* growth.

The inhibitory zones of ciprofloxacin (a positive control) were bigger than those of essential oils in all the culture media that were inoculated with *P. aeruginosa*. However, a reverse pattern was detected for all tested essential oils in the culture media of *E. coli*, wherein the inhibitory zones of essential oils for all populations were bigger than those of the positive control (ciprofloxacin). In addition, the inhibitory zones of all populations' essential oils were bigger than those of the tested antibiotic (vancomycin) for *S. aureus* bacterium, except those of Diyala, Saveh, and Urmia populations, whereas the inhibitory zones of vancomycin were bigger than those of essential oils.

## 4 DISCUSSION

We conducted a study to compare the composition of seed essential oils in different coriander populations from Iran and Iraq. The results showed that the essential oil profile was similar in all the populations tested. Monoterpene compounds made up more than 98% of the oils, with oxygenated monoterpenes being the most prevalent, followed by monoterpene hydrocarbons in a ratio of over 3:1, in almost all the populations. In addition, a small percentage of other compounds, such as hydrocarbons and aldehydes, were also detected. These findings are consistent with earlier studies conducted by Bhuiyan et al. (2009), Pande et al. (2010), Sriti et al. (2011). The percentage of monoterpene‐class compounds varied widely among the populations we examined, as well as those reported in previous studies (Mandal & Mandal, 2015). Essential oil composition of plants is known to be affected by various factors such as extraction method, cultivation, environmental, genetic, and ontogenetic parameters, according to several former investigations (Ebrahimi et al., 2010; Mohammadi & Saharkhiz, 2011; Sriti et al., 2011). We used the same extraction method to extract essential oils from mature seeds in all populations, so the observed variations in the populations were mainly due to cultivation method, environmental, and genetic parameters. The evaluated populations were cultivated under diverse ecological parameters in Iraq and Iran countries in terms of soil type, temperature, rainfall, and geographic coordinates (Zohary, 1973). Therefore, environmental parameters are considered one of the main reasons for these variations.

We did not find any sesquiterpene components in the essential oils obtained from the Iranian and Iraqi populations. However, previous investigations have detected trace amounts of sesquiterpene compounds in coriander seed oils. It appears that the environmental conditions of seed storage and seed growth stage significantly impact the presence of sesquiterpenes in coriander essential oil. For instance, Wahba et al. (2020) indicated that seed storage temperature strongly affects the percentages of monoterpenes and sesquiterpenes in coriander seed essential oil. Similarly, Msaada et al. (2009) found different amounts of sesquiterpene compounds in the essential oil of immature coriander seeds, while these compounds disappeared in ripe seeds. The major oil compounds detected among the evaluated populations were linalool (63%–74%), α‐pinene (8%–13%), γ‐terpinene (4%–10%), and geranyl acetate (2%–5%). These findings are consistent with Marangoni and de Moura (2011), who registered the major oil constituents as linalool (58.0%–80.3%), γ‐terpinene (0.3%–11.2%), α‐pinene (0.2%–10.9%), and geranyl acetate (0.2%–5.4%) in varying percentages, based on available references. Environmental factors and growth stages are believed to be the reasons for the observed differences in the percentages of these compounds. Pande et al. (2010) discovered that the composition of essential oils in coriander seeds varies during different stages of maturity. Specifically, the percentage of linalool changed during seed development in the Indian population. Mandal and Mandal (2015) also found that the amount of linalool varied by more than 1.5 times among coriander populations harvested from different regions of the world. Daniele et al. (2019) suggested that essential oil diversity within a species is heavily influenced by climate, soil type, period of collection, water availability, and nutrition. According to Wahba et al. (2020), the amount of linalool decreases with long‐term seed storage or exposure to cold stress.

All of the evaluated populations were found to be linalool chemotype based on their chemical compound analysis. However, they were grouped into four clusters during the clustering analyses. There was significant variation observed among the Iranian and Iraqi populations, and in some cases, populations from each country did not group together closely. Similar results have been reported in previous studies that investigated genetic molecular (Melo et al., 2011), morphological, and agricultural variables (Awas et al., 2016; Choudhary et al., 2022; Li et al., 2023).

We conducted a study on the antibacterial properties of essential oils extracted from plants against three types of bacteria – *P. aeruginosa*, *E. coli*, and *S. aureus*. The results indicated that the effectiveness of the essential oils was dependent on the concentration used; only oils at a 100% concentration showed any biological activity. Similar results were reported by Mandal and Mandal (2015), who found that the photogenic activity of coriander seed essential oil was dependent on its concentration.

The study found that the essential oils from the Baghdad population had the highest growth inhibitory effect against the *P. aeruginosa* bacterium, with a larger inhibitory zone than those reported in previous studies for other populations (Teshale et al., 2013). However, the essential oils from the Urmia, Maysan, and Diyala populations had no growth inhibitory effect on this bacterium. *P. aeruginosa* is an opportunistic bacterium that can cause bacterial infections and has become highly resistant to the most effective antibiotics. This multi‐drug‐resistant pathogen is a major challenge to public health worldwide due to its high morbidity and mortality rates (Alkhulaifi & Mohammed, 2023). The essential oils from the Baghdad population contained the highest amounts of camphor, terpinen‐4‐ol, and α‐terpineol, which may be responsible for the growth inhibitory effect against *P. aeruginosa*. Similar results were reported by Kotan et al. (2007), who found antibacterial activity of terpinen‐4‐ol and α‐terpineol against *P. aeruginosa*.

Our findings explored that *P. aeruginosa* bacteria were more susceptible to ciprofloxacin than the coriander essential oils of the evaluated Iranian and Iraqi populations. Duartea et al. (2018) indicated that the essential oil obtained from *C. sativum* seeds has good activity against some human pathogenic bacteria, such as *P. aeruginosa*. In addition, it can improve the antibacterial activity of ciprofloxacin (Duarte et al., 2012), and seems it can play a synergistic role in the inhabitation growth of drug resistance strains of *P. aeruginosa*.

The essential oil of the Saveh population had the highest growth inhibitory effect against *E. coli*. However, this biological activity was lower than that detected for *E. coli* in previous research (Teshale et al., 2013). In addition, all of the examined essential oils explored various degrees of growth inhibitory effect against this bacterium.

According to Ghanem and Haddadin (2018), *E. coli* is considered a diverse bacterium in the sense of virulence and pathogenicity. This bacterium is widely observed in open environments and can easily spread to cause risks in human and other animals. Some strains of *E. coli* isolated from household and hospital environments revealed a high resistance rate to diverse classes of antibiotics. Additionally, no significant difference was detected between bacteria isolated from two environments.

Meanwhile, the inhibitory effect of all tested essential oils was stronger than that of ciprofloxacin for *E. coli* bacteria. Fasugba et al. (2015) suggested that ciprofloxacin resistance in *E. coli* urinary tract infections has increased. Therefore, the application of this antibiotic as an empirical therapy for urinary tract infections should be reconsidered. Policy restrictions on ciprofloxacin use must be increased in various parts of the world. Our findings indicated that coriander essential oil can be used as a strong growth inhibitor in plant‐derived materials instead of ciprofloxacin.

The essential oils from the Dezful and Lorestan populations showed the highest inhibitory effect against *S. aureus*. Additionally, all tested oils exhibited varying levels of growth inhibitory effects against the bacteria. *S. aureus* is considered a significant pathogen in hospitals and open systems, leading to several infectious diseases. In recent times, drug‐resistant strains of *S. aureus* have emerged, making it more challenging to treat them clinically (Guo et al., 2020). The populations in Dezful and Lorestan exhibited the highest percentages of linalool, which is a compound found in coriander seeds. Additionally, nerol, a compound that is not commonly present in coriander seed essential oil, was detected in some populations. It has been suggested by Maleki et al. (2021) that certain monoterpenoid alcohol compounds, including linalool and nerol, possess strong antibacterial properties due to the presence of double bonds in the structure of their molecules. Hence, it is likely that the high percentages of linalool and nerol found in the essential oils of the Dezful and Lorestan populations contribute to their antibacterial efficacy against *S. aureus*.

Essential oils of most populations had a stronger inhibitory effect on *S. aureus* bacteria growth than vancomycin. Cong et al. (2020) suggested that infections caused by methicillin‐resistant *S. aureus* become a global threat to human health. In this sense, some antibiotics, such as vancomycin, are the first‐line drugs for the treatment of these infections. Meanwhile, diverse isolates of this bacterium with complete resistance to vancomycin have been detected in recent years. However, our findings indicated that the essential oils extracted from all populations have enough potential to exhibit antibacterial activity against *S. aureus* bacteria; therefore, the seed‐extracted oils of various coriander populations are good candidates to inhibit the growth of these bacteria in foods and treat infectious diseases caused by *S. aureus*. Plant‐derived materials explore a great potential to employ either antimicrobials or antibiotic‐resistance modifiers (Al Sheikh et al., 2020).

According to our results, Gram‐positive bacteria were more susceptible to coriander seed essential oil than Gram‐negative ones. Meanwhile, a reverse pattern was reported by Silva et al. (2011). Additionally, *P. aeruginosa* was more resistant to coriander seed essential oil than other bacteria. A similar finding was detected in the earlier study (Silva et al., 2011).

## 5 CONCLUSION

The study compared the composition of essential oils in coriander seeds from nine populations in Iran and Iraq using GC and GC/MS apparatus for the first time. The results showed that a significant portion of the oils in all populations were oxygenated hydrocarbons, with the same components being detected as the major constituents of the essential oils. Linalool was found to be the main component in all cases, but its percentage varied widely among the populations. Clustering analyses showed that Iranian and Iraqi coriander populations did not group closely. All the populations were found to be linalool chemotypes, characterized by a specific chemical compound. The essential oils exhibited antibacterial activity against the tested bacteria when used in 100% concentration, with the highest activity being detected against *S. aureus*, where the essential oils of all populations inhibited the growth of these bacteria. These findings provide important information for coriander breeders, growers, and researchers seeking to enhance coriander production and use. Further research is needed to explore the genetic factors affecting essential oil composition, biological activity and to develop strategies for increasing essential oil quality and yield. Such targeted research can optimize the utilization of coriander seed essential oil and minimize waste. Additionally, examining the specific antibacterial applications of each population could provide valuable insights for targeted utilization.

## AUTHOR CONTRIBUTIONS

**Seyed Mehdi Talebi:** Conceptualization (equal); data curation (equal); formal analysis (equal); project administration (equal); software (equal); writing – original draft (equal). **Abbas Naser:** Investigation (equal); methodology (equal); software (equal); visualization (equal); writing – original draft (equal). **Mansour Ghorbanpour:** Resources (equal); software (equal); writing – review and editing (equal).

## CONFLICT OF INTEREST STATEMENT

The authors declare no competing interests.

## ETHICS STATEMENT

All methods performed in this study, including the collection of plant materials, were in compliance with the relevant institutional, national, and international guidelines and legislation.

## STATEMENT OF COMPLIANCE

The authors confirm that all the experimental research and field studies, including the collection of plant material, complied with relevant institutional, national, and international guidelines and legislation.

## STATEMENT ON EXPERIMENTAL RESEARCH AND FIELD STUDIES ON PLANTS

The growing plants sampled comply with relevant institutional, national, and international guidelines and domestic legislation of Iran and Iraq.

## Data Availability

All the data generated or analyzed during the current study were included in the manuscript. The raw data are available from the corresponding author on reasonable request.
